# Case Report: Septic Pericarditis With *Achromobacter xyloxidans* in an Immunosuppressed Dog

**DOI:** 10.3389/fvets.2022.884654

**Published:** 2022-05-18

**Authors:** Kristina M. Pascutti, Jacqueline K. Dolan, Lauren T. Porter, Shir Gilor, Autumn N. Harris

**Affiliations:** ^1^Department of Small Animal Clinical Sciences, University of Florida, College of Veterinary Medicine, Gainesville, FL, United States; ^2^Department of Comparative, Diagnostics and Population Medicine, Gainesville, FL, United States; ^3^Division of Nephrology, Hypertension and Renal Transplantation, University of Florida, College of Medicine, Gainesville, FL, United States

**Keywords:** canine, bacterial, pericarditis, Apoquel, pyothorax

## Abstract

A 5-year-old female spayed French Bulldog presented for anorexia and increased respiratory rate. On presentation, she was dyspneic with stridor and increased bronchovesicular sounds. Point-of-care ultrasound identified pericardial effusion. Thoracic radiographs identified pleural effusion, a wide cranial mediastinum, and multifocal unstructured interstitial pulmonary opacities. Bloodwork revealed a moderate leukocytosis characterized by a mature neutrophilia with a left shift, hypoalbuminemia, mildly increased alkaline phosphatase activity, and moderate hypokalemia. Thoracic CT findings revealed moderate pericardial and bilateral pleural effusion, mediastinal effusion, and moderate cranial mediastinal lymphadenopathy. Diagnostic thoracocentesis and pericardiocentesis revealed septic exudates with bacilli. Two days later, a median sternotomy and pericardiectomy were performed. Aerobic cultures of the effusions grew *Achromobacter xylosoxidans* ss *deitrificans*. The patient was treated with Amoxicillin-clavulanate and enrofloxacin for 12 weeks and clinically fully recovered. *Achromobacter xylosoxidans* has not been reported as a cause of purulent pericarditis and pyothorax in a dog. Uniquely, this patient is suspected of developing this infection secondary to immunosuppression.

## Introduction

In the veterinary literature, septic pericardial effusion is a rarely described form of canine pericardial disease, with bacterial infections leading to septic pericarditis in <10% of all cases of pericardial effusion (1). The most common causes of pericardial effusion include neoplasia, idiopathic, and a left atrial tear secondary to degenerative valve disease (1–3). Septic pericarditis is typically associated with dog fight wounds, migrating foreign bodies, an extension of local disease, or hematogenous spread of infection (4–10). Infectious organisms that have been implicated in septic pericarditis in dogs include *Staphylococcus, Streptococcus, Pseudomonas*, Nocardia, Bacteriodes, *Pasturella multocida, Actinomyces, Acinetobacter*, aspergillosis, *Candida albicans*, coccidomycosis, and dirofilariasis (1, 4, 5, 7–10). The general treatment recommendations for septic pericarditis include the administration of appropriate antibiotics or antifungals, removing the pericardial fluid *via* pericardiocentesis, and subtotal pericardectomy (2).

*Achromobacter xylosoxidans* is an emerging pathogen in people, most commonly associated with healthcare-associated infections and immunocompromised patients (11, 12). This bacterium is known for its resistance mechanisms leading to high morbidity and mortality in people (11, 13). However, its significance in dogs and cats is unknown.

This report describes the clinical features and outcome *of Achromobacter xyloxidans* purulent pericarditis and pyothorax in an immunosuppressed 5-year-old female spayed French Bulldog that was managed with medical stabilization and subtotal pericardiectomy. To the authors' knowledge, this is the first report in the veterinary literature of *A. xylosoxidans* leading to purulent pericarditis and a pyothorax in a dog.

## Case Description

A 5-year-old female spayed French Bulldog, weighing 8.6 kg, was evaluated at the University of Florida Small Animal Hospital (UFSAH) for acute increased respiratory effort and anorexia. Pertinent past medical history included chronic small bowel diarrhea and vomiting diagnosed as inflammatory bowel disease based on gastrointestinal biopsies and managed with a hydrolyzed diet (Science Diet Z/D; Hill's Pet Nutrition, USA) and immunosuppressive doses of prednisone (1 mg/kg PO BID; predniSONE Intensol, USA). The dog also had a history of atopy managed with oclacitinib (0.55 mg/kg, PO BID; Zoetis, USA). At the time of presentation, the dog was still receiving these medications.

The dog was bright, alert, and responsive on initial physical examination. Her vitals at presentation revealed that she was normothermic with a rectal temperature of 102.1°F, a heart rate of 96 beats per minutes, and mildly tachypnic (44 breaths per minute) with increased effort. Her mucous membranes were pink, with a capillary refill time of <2 s. Cardiothoracic auscultation revealed mildly increased bronchovesicular sounds in her cranioventral lung fields bilaterally. Mild stridor was noted. Abnormalities were not detected on the remainder of the exam. Clinical pathology abnormalities included a moderate leukocytosis (32,650 cells/μl, range 5,000–13,000 cells/μl) characterized by a moderate neutrophilia (27,000 cells/μl, range 2,700–8,900 cells/μl) with a left shift (4,700 band neutrophil cells/μl), and a lymphopenia (600 cells/μl, range 900–3400 cells/μl). White blood cell morphology revealed a moderate toxic change to many neutrophils. There was moderate hyperfibrinogenemia (0.8 g/dl, range 0.1–0.4 g/dl). There was a mildly increased alkaline phosphatase activity (201 U/L, range 7–116 U/L), mild hypoalbuminemia (2.55 g/dl, range 2.62–3.91 g/dl), moderate hypokalemia at (2.9 mEq/L, range 3.8–5.0 mEq/L), and marginal hypochloremia (106.3 mEq/L, range 107.8–117.1 mEq/L). Urinalysis (*via* cystocentesis) revealed 4+ sulfosalicylic acid protein, 0–3 white blood cells/hpf, 0–5 red blood cells/hpf, and a few bacilli. This urine sample was submitted for aerobic culture. Thoracic radiographs revealed multiple, patchy, ill-defined increases in soft tissue opacity (up to 2.5 cm in diameter) that partially obscured the pulmonary vasculature in the right caudal lung lobe along with air bronchograms in the right cranial lung lobe. In addition, there was moderate widening with soft tissue opacity in the cranial mediastinum. A point of care thoracic ultrasound identified a small volume of pericardial effusion. An echocardiogram was then performed which confirmed the small volume pericardial effusion and determined it was not resulting in hemodynamic compromise. No discrete mass as the cause of the pericardial effusion was identified on echocardiogram. Due to the small volume of pericardial effusion, it was elected to hospitalize the patient overnight and pursue additional diagnostics the following day. The dog was hospitalized, and initial treatment started with intravenous fluid therapy using Lactated Ringer's solution (Dechra Veterinary Products, USA), at 2.5 ml/kg/h supplemented with 0.2 mEq/kg/h of KCl (Baxter Healthcare Corporation, USA) supplementation. Since the patient was stable, it was elected to hold on initiation of empiric antimicrobial therapy until further diagnostics were performed unless the clinical status changed overnight.

**Figure 1:**
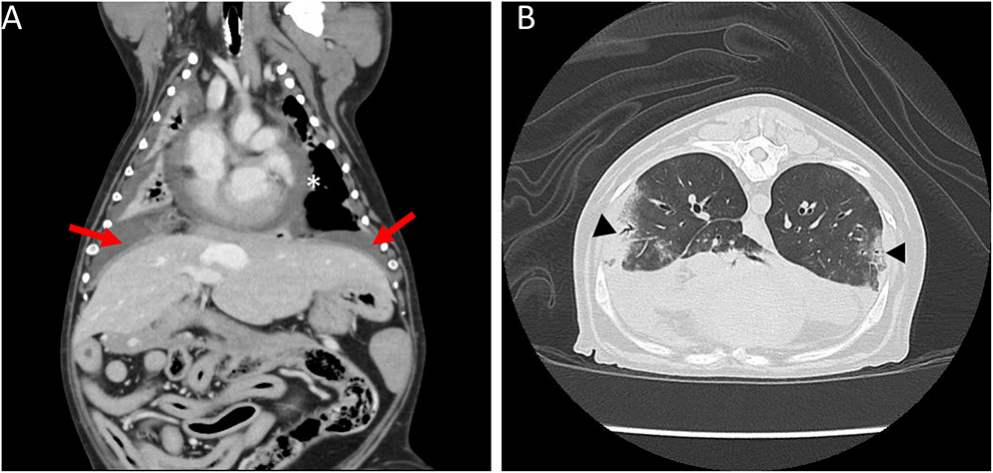
Coronal and sagittal computed tomography (CT) images of the thorax. **(A)** Moderate bilateral pleural effusion (arrows) and moderate pericardial effusion (asterisk). **(B)** Multifocal mixed unstructured interstitial to coalescing alveolar pulmonary pattern (triangles).

**Figure 2:**
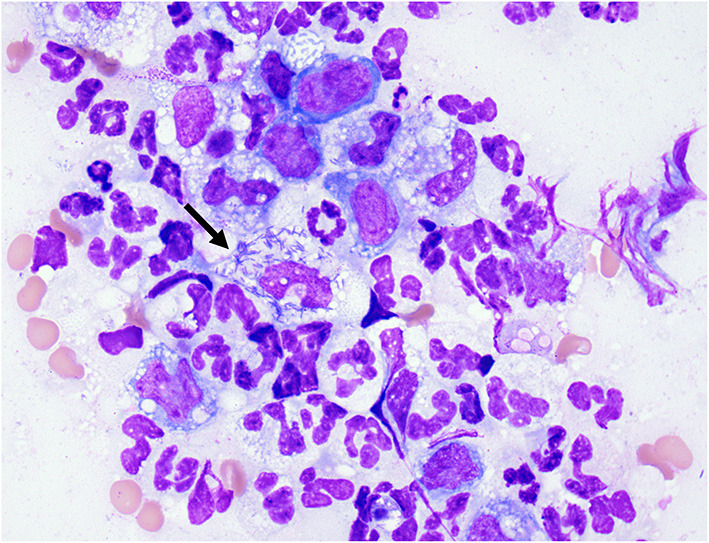
Cytology of pleural fluid. Photomicrograph of pleural effusion. Mixed inflammation, neutrophils and variably vacuolated macrophages. Neutrophil (arrow) with phagocytosed intracellular thin bacilli. Wright-Giemsa stain 100× oil objective.

The following day, the dog was anesthetized for a thoracic computed tomography (CT) and bronchoscopy with bronchoalveolar lavage (BAL). Thoracic CT with intravenous contrast revealed moderate pericardial and bilateral pleural effusion, mediastinal effusion, a mixed unstructured interstitial to coalescing pulmonary alveolar pattern affecting the dorsal part of the lung lobes, and moderate cranial mediastinal lymphadenopathy (2.5 cm in diameter; Figure 1). Bronchoscopy revealed diffusely hyperemic mucosa and with mild amount of mucous in the trachea. The BAL cytology revealed moderate neutrophilic inflammation with a WBC count of 2,000 cells/μl. Based on a 200 cell count, the leukocyte differential was 92% neutrophils and 8% macrophages. Diagnostic thoracocentesis and pericardiocentesis were performed. Grossly the pericardial fluid appeared red and cloudy. Pericardial effusion cytology revealed an exudate with a WBC count of 114,800 cells/μl and a total protein of 2.8 g/dl. Based on a 200 cell count, the leukocyte differential was 93% degenerate neutrophils and 6% macrophages. The pleural effusion appeared turbid and orange, grossly. Pleural effusion cytology revealed a septic exudate with a WBC count of 117,500 cells/μl and a total protein of 3.7 g/dl. Based on a 200 cell count, the leukocyte differential was 79% degenerate neutrophils and 19% macrophages with intracellular aggregates of thin bacilli seen (Figure 2). Both the pleural and pericardial effusions were submitted for aerobic and anaerobic cultures. In addition, the patient had aerobic and anaerobic blood cultures collected from two different sites, 1 h apart (cephalic vein and saphenous vein). Following these diagnostics, empiric antimicrobial therapy was initiated with clindamycin 15 mg/kg IV q12 hours (Zoetis, USA) and enrofloxacin 10 mg/kg IV q24 hours (Bayer, USA) while the patient awaited surgery. The decision to delay surgery to the following day was multifactorial including being after-hours due to other emergencies and her stable clinical status at that time.

A median sternotomy, subtotal pericardiectomy, and thoracic exploratory were performed 2 days after initial presentation. All lung lobes were palpated and examined during the procedure, with no overt abnormalities found. There was approximately 10 ml of serosanguinous fluid within the pericardial space. A subtotal pericardiectomy was performed with a 7 cm × 4 cm pericardial tissue sample collected for histopathology. Aerobic and anaerobic cultures were obtained of the pericardial and pleural effusion, pericardium, and mediastinum intraoperatively. No other gross abnormalities were noted. At the time of surgery, bilateral thoracostomy tubes (12 Fr, Mila International, USA) were placed and secured using a finger trap suture pattern. For the first 24 h post-operative period, the thoracostomy tubes were flushed every 4 h with 10–20 ml of sterile saline per chest tube. Treatment was continued with enrofloxacin (10 mg/kg IV SID; Bayer, USA) and clindamycin (15 mg/kg IV BID; Zoetis, USA) pending culture results. A single 1 mg/kg prednisone equivalent of dexamethasone SP (Covetrus, USA) was administered IV on the day of surgery and then discontinued. Following surgery, oclacitnib was also discontinued. The right chest tube was removed within 24 h due to no fluid production, and the left chest tube was removed 2 days post-operative when drain production was <2 ml/kg/day.

After 3 days, the urine aerobic culture and blood anaerobic and aerobic cultures had no bacterial growth. The effusion samples, pericardium, and mediastinum tissues grew *Achromobacter xylosoxidans* ss *denitrificans* on aerobic culture. The susceptibility panel showed bacterial resistance to cephalosporins and sensitivity to fluoroquinolones and Amoxicillin-clavulanate (Table 1). Based on the susceptibility panel, clindamycin and enrofloxacin were discontinued. The dog was started on Amoxicillin-clavulanate (14 mg/kg PO BID). She represented 24 h after discharge for tachypnea. No significant changes to her physical exam were found, and there was no recurrence of pleural or pericardial effusion. Since the only change at discharge had been to stop the enrofloxacin, she was restarted on enrofloxacin (11 mg/kg PO SID; Bayer, USA) in conjunction with the previously prescribed amoxicillin-clavulanate for 12 weeks. Recheck thoracic radiographs were performed 2 weeks, 1 month, and 3 months after surgery, which revealed markedly improved pleural effusion, normal pulmonary parenchyma, cardiovascular structures, and mediastinum. Clinically, the patient was doing well at home with no increased respiratory rate or effort and no gastrointestinal signs. At this time, her IBD was well managed on Science Diet Z/D, and her atopy was being managed with diphenhydramine (2.5 mg/kg PO BID; Johnson and Johnson, USA).

**Table 1:** Antimicrobial sensitivity for *Achromobacter xylosoxidans* ss *denitrificans* from pleural and pericardial effusion, pericardium, and mediastinal tissues.

**Antibiotic**	**MIC**	**Interpretation**
Amikacin	≤4	S
Amoxicillin/clavulanic acid	1	S
Ampicillin	8	NI
Cefalexin	4	NI
Cefazolin	8	R
Cefovecin	>8	NI
Cefpodoxime	>8	NI
Ceftazidime	≤4	S
Chloramphenicol	4	S
Doxycycline	2	NI
Enrofloxacin	≤0.12	S
Gentamicin	≤0.25	S
Imipenem	≤1	NI
Marbofloxacin	≤0.12	NI
Orbifloxacin	≤1	NI
Piperacillin/tazobactam	≤8	S
Pradofloxacin	≤0.25	NI
Tetracycline	≤4	NI
Trimethoprim/sulfamethoxazole	≤0.5	S

*S, sensitive; I, intermediate; R, resistant; NI, no interpretation exists according to CLSI standards*.

The patient was re-presented to UFSAH 7 months after the first surgery for increased respiratory rate and effort. On physical exam, she was intermittently tachypneic (50 breaths per minute) and tachycardic (180 beats per minute) with no other abnormalities noted. A point of care thoracic ultrasound revealed no recurrence of pleural or pericardial effusion. Thoracic radiographs were unchanged from previous diagnostic imaging. With no significant findings, a complete blood count, serum biochemistry, and venous blood gas were performed. She was hospitalized for 24 h for monitoring and received only Trazodone (5 mg/kg PO TID; Pragma Pharmaceuticals, USA), and she clinically improved. There was no determined cause for this episode of tachypnea. Since writing, the patient has not relapsed her infection at 12 months post-surgery.

## Discussion

This case is unique for multiple reasons. The dog's bacterial pericarditis did not appear to have been caused by a wound or migrating foreign body, as a most commonly described cause of septic pericarditis. It is possible that a migrating foreign body was the nidus for infection but was unable to be identified through advanced imaging and exploratory surgery. Given this dog's chronic history of skin disease, it is also possible that this dog acquired the infection through a skin infection given the bacterium's ubiquity in the environment. We propose that this dog developed an opportunistic infection secondary to immunosuppression from prednisone and oclacitinib. Immunosuppression has been linked to the development of opportunistic infections in both veterinary and human medicine (5, 11, 12, 14, 15). One other case report describes a dog that developed septic pericarditis with *C. albicans* and *Acinetobacter* 39 days following treatment initiation for immune-mediated hemolytic anemia with prednisolone and azathioprine (5). For the dog in this case report, it is unknown if a single drug predisposed her to develop this opportunistic infection, a combination, or another unknown factor. In a literature review, most canine cases with an opportunistic infection had been on multiple immunosuppressant medications an average of 69 days before presenting with infections (15–18). The dog reported here had been on prednisone 19 days and oclacitinib approximately 1,440 days before she was diagnosed with septic pericarditis. This raised concern that immunosuppression secondary to oclacitinib was a significant factor in the development of septic pericarditis in this dog. However, we cannot exclude other factors such as her immune system or sensitivity to prednisone as reasons for developing an infection in a relatively short period of time after initiating therapy with steroids compared to the prior reports.

Oclacitinib (Apoquel) is a Janus kinase inhibitor prescribed to help relieve pruritis associated with various dermatological conditions. Its use has been associated with developing multiple diseases, including demodicosis, skin and ear infections, and urinary tract infections, suggesting it may have some immunomodulatory or immunosuppressive effects (19–21). A recent study evaluated the impact of oclacitinib on canine T cell proliferation and cytokine production (22). This study found that at high doses defined as 3–4 mg/kg q12 hours, oclacitinib had strong immunosuppressive effects resulting in reduced interleukin-2, interleukin-15, interferon-gamma, and interleukin-18a concentrations (22). However, at the recommended dose of 0.4–0.6 mg/kg, it failed to demonstrate any strong immunosuppressive effects (22). The dog in this case report was receiving the recommended dose of oclacitinib at the time of presentation. Two additional studies evaluating the long-term effects of oclacitinib in dogs showed the most common adverse effects were diarrhea, vomiting, urinary tract infections, pyoderma, and otitis externa (19, 23). However, they also reported a Labrador retriever that developed abdominal and pleural effusions after 450 days of oclacitinib administration (23). The cause of the effusions was not specified in the report. In addition, they reported a Pug that developed bronchopneumonia in which the bacterial species was not reported (19). Finally, they showed neoplasia developing in 16 dogs included in the study (19). These findings may support that oclacitinib does have some immunomodulatory effects.

*Achromobacter xylosoxidans* is an aerobic, motile, nonfermenting gram-negative rod ubiquitous in the environment. In humans, this bacterium is commonly associated with nosocomial infections in patients with immunosuppression or hematological malignancies (11, 12). *Achromobacter xylosoxidans* infections most commonly present as bacteremia (11, 24). There are many reports of this infection being traced back to indwelling devices such as intravenous catheters. However, one study found a low prevalence of catheter-associated infections occurring in 13/52 cases (25%) (12). *Achromobacter xylosoxidans* has also been reported as the cause of infection in human patients with meningitis, urinary tract infection, an abscess, osteomyelitis, peritonitis, pneumonia, corneal ulcer, and prosthetic valves (11, 12). Furthermore, it has been reported that approximately 11% of cystic fibrosis patients have an infection involving *A. xylosoxidans* which tends to be a community-acquired infection (25).

Infection with *A. xylosoxidans* has not been well reported in veterinary medicine, so its significance as a pathogen is unknown. This organism may have been underreported in veterinary medicine from not being recognized as it is easily confused with other gram-negative bacteria such as *Pseudomonas aeruginosa* (13). This is because both organisms are aerobic, gram-negative, non-spore forming rods that are both oxidase and catalase positive (13, 26). To distinguish between the two microbes, the flagella must be identified (26). *Achromobacter xylosoxidans* have a peritrichous flagella and *Pseudomonas* have a single polar flagellum (26). There are a few case reports in veterinary patients to the author's knowledge describing nosocomial infections with this bacterium secondary to surgical interventions, and one reported urinary tract infection in a cat (27–30). In veterinary medicine, this is the first case report of *A. xylosoxidans* causing purulent pericarditis and pyothorax.

*Achromobacter xylosoxidans* infections are challenging to treat due to the reduced effectiveness of a wide range of antimicrobial therapies. *Achromobacter xylosoxidans* inherent resistance mechanisms, including multidrug efflux pumps and beta-lactamases, have been associated with high morbidity and mortality in people (11, 13, 24). *Achromobacter xylosoxidans* are intrinsically resistant to aminoglycosides and 1st and 2nd generation cephalosporins; however, resistance to carbapenems is increasing (11, 24). The bacterium cultured from our patient had a resistance to early generation cephalosporins, consistent with what is reported in human literature (11, 24). Our patient was treated with a potentiated penicillin and a fluoroquinolone for 12 weeks with no evidence of a relapse.

The decision to treat for 12 weeks with antimicrobials was multifactorial. Part of the decision for a prolonged course of antimicrobial therapy was based on concerns that this dog was immunocompromised, whether secondary to the drugs that were discontinued or an underlying immune disorder. In addition, there is currently no consensus on how long to medically manage a pyothorax or mediastinitis in either the human or veterinary literature. The mediastinum in this dog was not surgically explored. Since the culture of the mediastinal tissue was positive for bacteria, it was suspected that the infection may be throughout the mediastinum. Using a traditional approach, antibiotics were continued with the goal of treating 2 weeks past radiographic resolution of the mediastinal changes and pleural effusion. However, this patient continued to have an abnormally widened mediastinum with increased soft tissue opacity on thoracic radiographs which may reflect a breed-related anatomic variation. Antibiotics were discontinued when the dog had been doing clinically well and bloodwork was within normal limits.

As previously mentioned, the dog reported here had been receiving prednisone at a 2 mg/kg/day dose for 19 days prior to presentation. There is currently no consensus in the veterinary literature as to when to taper prednisone vs. abruptly discontinue the medication. In general, for most conditions we treat with prolonged courses of steroids, part of the rationale for tapering is to monitor for a relapse of the underlying disease. In this case, the dog had IBD which was considered non-life-threatening compared to the infection. The other rationale for tapering steroids is to allow for recovery of the hypothalamic-pituitary-adrenal axis. In the human literature, the recommendation is to do a taper course of steroids if the patient has been on high doses for longer than 2 weeks to help decrease the risk of adverse effects such as hypotension and exacerbation of gastrointestinal signs (31). The decision for the patient in this study was made to give a 1 mg/kg prednisone equivalent of dexamethasone SP the day of surgery to assist with the added stressors of the procedure followed by monitoring the patient for evidence of adrenal insufficiency in the post-operative period. Our assessment for this patient had been that the risks of life-threatening adrenal insufficiency were low compared to the risks for continued immunosuppression and poor post-operative wound healing had glucocorticoids been continued.

This patient had multiple pulmonary changes identified on thoracic CT. One finding was a ventrally distributed alveolar pattern causing effacement of the local pulmonary vasculature and air bronchograms. In conjunction with this finding, there was a moderate amount of fluid-attenuating material wthin the pleural space consistent with effusion causing round and retraction of the ventral aspect of all lung lobes. These pulmonary changes are likely explained by atelectasis secondary to the pleural effusion. However, other differentials such as bronchopneumonia or primary tumor can't be excluded without histopathology. During the thoracotomy, the lungs appeared and palpated normal with no overt abnormalities suggesting a mass or tumor is less likely the cause of those changes. In addition to the ventral alveolar pattern, CT demonstrated a multifocal mixed unstructured to coalescing alveolar pulmonary pattern in the dorsal part of the lung lobes. In this case, it would be reasonable to hypothesize that those changes are secondary to a bronchopneumonia caused by *A. xylosoxidans*. Case reports in humans with pneumonia secondary to *A. xylosoxidans* demonstrate multifocal pulmonary infiltrates and consolidation as well as pleural thickening on CT imaging similar to our case (32–34).

This case is unique in the veterinary literature. The clinical findings document both the first case of *A. xylosoxidans* causing septic pericarditis and pyothorax in an immunosuppressed dog with the absence of a documented migrating foreign object or penetrating wound trauma. When cultured, investigation into nosocomial sources of infection or immunosuppression should be considered. The use of oclacitinib and immunosuppressive doses of glucocorticoids should be used cautiously, given concern for developing opportunist infections.

## Data Availability Statement

The original contributions presented in the study are included in the article/supplementary material, further inquiries can be directed to the corresponding author.

## Author Contributions

AH and KP: conception and design of report. JD and SG: prepared figure. KP: drafted manuscript. KP, AH, JD, SG, and LP: edited and revised the manuscript. All authors approved the final version of the manuscript.

## Conflict of Interest

The authors declare that the research was conducted in the absence of any commercial or financial relationships that could be construed as a potential conflict of interest.

## Publisher's Note

All claims expressed in this article are solely those of the authors and do not necessarily represent those of their affiliated organizations, or those of the publisher, the editors and the reviewers. Any product that may be evaluated in this article, or claim that may be made by its manufacturer, is not guaranteed or endorsed by the publisher.
